# Progress in Psychiatry

**Published:** 1901-10-26

**Authors:** 


### PROGRESS IN PSYCHIATRY.

(*Continued from page* 51.)

**Duration and Terminations of Puerperal Insanity.** The more grave and acute septic cases, attended by marked delirium and fever, with dry tongue, soudes on the lips and teeth, offensive breath, and constipation, are of short duration and end in death. Others improve after removal of the sources of sepsis (clots and membranes retained in the uterus, pus in the uterus and vagina), and repeated douching out of the uterus and vagina with antiseptic solutions (boracic acid, carbolic acid, izal, etc.). Curettage of the endometrium is needed in some cases to remove all septic and putrefying material. The occurrence of marked cerebral exhaustion is of bad prognosis, as the patient lives only a few days, or, where recovery takes place, the process of restoration to health is slower than in septic cases. The average duration of puerperal insanity is eight months (Jelly), and in the majority of these recovery sets in during the third or fourth month. In those cases which show a tendency to periodicity, and in which slight stresses co-operate with marked hereditary predisposition, the signs of recovery show themselves earlier, *i.e.* during the second or third month of illness, and generally speaking, cases of earlier and more abrupt onset tend to show signs of returning health earlier than cases of gradual and late onset. According to Hoppe, the average duration of the hallucinatory confusional form of puerperal insanity is 9½ months, that of the melancholic form 13 months, and that of the hysterical form 19½ months. Relapses not infrequently occurred during convalescence (Jones),[12] and were of bad import. Special points to watch and guard against were suicide, impulses to infanticide, obscene and erotic manifestations, and bodily injuries sustainable from excessive restlessness and movement. The subsidence of acute symptoms in some patients was marked by a tendency to apathy and stupor. This termination should be prevented by brightening and changing the surroundings of the patient, and stimulating her by amusements and the presence and conversation of husband and friends. In a general way treatment consisted of light liquid diet freely and frequently administered (by the œsophageal or nasal feeding-tube if necessary). This was needed to combat the profound exhaustion present. Sleep and rest should be secured by the use of hypnotics such as sulphonal, paraldehyde, or the combination of chloral and bromides. Anæmia and cardiac feebleness should be combated by iron, hæmoglobin preparations, digitalis, and strychnine. Electric baths were especially found useful in stuporous states (Jones). The bowels should be freely opened with castor oil, cascara sagrada, calomel, or croton oil, and kept open regularly. Blue pill and black draught were good for this purpose. Daily catheterisation of the bladder was necessary in those cases that suffered from inability to empty the bladder. Small amounts of alcohol might be mixed with the liquid food when it was thought a stimulant was necessary. The terminations of puerperal insanity may be various. Clouston[13] reports that 8·33 of his cases of puerperal insanity terminated fatally, McLeod[14] gives the ratio of mortality as 9 per cent., and Menzies[15] as 10 per cent. Bevan Lewis[16] gives the ratio as 8·5 per cent. The prognosis as to recovery from a first attack is good. Thus Hoche found that 40 per cent. of the total cases collected by him (98 cases of puerperal insanity, 24 of pregnancy, and 89 of lactation) recovered, but if gestational and lactational cases were excluded the ratio of recovery in puerperal insanity would be higher. McLeod reports that 77 per cent. recovered, while Clouston gives 55 per cent. of puerperal cases as recovered, and 27 per cent. as improved. Menzies gives the ratio of recoveries as 43 per cent, and observes that 46½ per cent. become chronic and incurable. Lewis gives 83 per cent. as recovered, and 5·7 per cent. as becoming chronic. The tendency to recurrence, however, is strong with subsequent confinements. Thus of Dr. Jelly's 200 cases of puerperal insanity 30 had had more than one attack. "One woman was admitted to a hospital for the insane in 1870 with her first attack, and in 1899 in her seventeenth attack." Dr. Jones records a similar case of recurrence. After a second or third attack the prognosis is bad as a tendency to chronicity or recurrence is established. The occurrence of the menopause generally brings with it chronic insanity and dementia in women who have had several attacks of puerperal insanity if that terminus has not already been attained. Recurrence is particularly noticeable among women of the poorer classes, who suckled their children on returning home from the asylum. The return to a normal mental condition, if such is to be the result, is a gradual one. The hallucinations slowly subside and are not succeeded by delusions, the physical condition improves, food is taken more abundantly, the intervals of comparative lucidity become longer and longer, and the menstrual function is re-established. The patient then gradually returns to her normal mental state. In other cases, with a more tardy recovery, the apparent convalescence is marked by depression and mutism. This form of convalescence is apparent rather than real, for although acute hallucinations and excitement have subsided, a condition of dream-like mental confusion with vague delusions and fears prevail, comparable to the so-called delusional stupor. It is almost impossible to get the patients to converse, or to take any interest in persons or surroundings. They are unable to feed themselves or attend to their wants, and demand constant care and nursing (feeding, clothing, etc.). The progress towards eventual recovery is slow in some, while in others the progress is towards chronicity, with permanent development of delusions, principally of a persecutory and sometimes of an erotic type. Physical health may be now regained, weight and appetite may increase, but the mental condition is that of chronic insanity, gradually lapsing to dementia. The general facts regarding prognosis and termination may be summed up as follows :—Of ten women who develop puerperal insanity for the first time, one dies, two recover partially from the acute stage, have intermissions, and relapse into chronic insanity, and seven completely recover. Of the seven who recover, the majority (say five) develop puerperal (or other) insanity with subsequent childbirths (or other stresses), *and but two of the original ten remain wholly sane for the rest of their lives.*

The insanity of lactation is essentially one of inanition, and develops during or after the third month of lactation. The following causes contribute to it: the exhaustion of the mother from suckling, the frequent interruption of the mother's sleep at night for nursing the babe, and the after effects of labour and accompanying hæmorrhage which have not been perfectly recovered from. The changes incidental to uterine involution also play a minor part in loading the blood with metabolic products. A combination of these causes is sufficient in physically weak women who have insufficient food and rest, to produce mental breakdown. Any hereditary taint and depressing surroundings will have their share in accelerating the development of insanity. The "insanity of lactation" is thus essentially an exhaustion-psychosis. It tends to depression rather than excitement, and its superficial symptoms are those of melancholia rather than mania. Where septicæmia from pelvic abscess or mastitis is present the symptoms tend to approximate to those of a febrile delirium with pyrexia and acute mental confusion. The patient is usually pale or waxy-looking with anæmia, and has suffered for weeks from insomnia and loss of appetite. She now develops hallucinations, hears "voices," or sees terrifying objects. She conceives sudden and excessive terrors, and under the influence of them may slay her child, or attempt violence to herself or to others. Excitement is thus present in about two-thirds of all such cases, and depression (apathy or stupor and confusion) in about one-third. True melancholia seldom occurs. The prognosis is bad, as in rather more than 50 per cent. recovery is partial and the tendency is towards dementia.

[12] Lancet, Aug. 17, 1901. [13] Clin. Lectures on Mental Diseases. [14] Brit. Med. Journ., 1886. [15] Journ. of Mental Science, and Amer. Journ. of Insanity, 1893. [16] Text-book of Mental Diseases.

